# Paired Burst Stimulation Causes GABA_A_ Receptor-Dependent Spike Firing Facilitation in CA1 of Rat Hippocampal Slices

**DOI:** 10.3389/fncel.2016.00009

**Published:** 2016-01-29

**Authors:** Takashi Tominaga, Yoko Tominaga

**Affiliations:** Laboratory for Neural Circuit Systems, Institute of Neuroscience, Tokushima Bunri UniversitySanuki, Japan

**Keywords:** hippocampus, theta, voltage-sensitive dye, GABA_A_ receptor

## Abstract

The theta oscillation (4–8 Hz) is a pivotal form of oscillatory activity in the hippocampus that is intermittently concurrent with gamma (25–100 Hz) burst events. In *in vitro* preparation, a stimulation protocol that mimics the theta oscillation, theta burst stimulation (TBS), is used to induce long-term potentiation. Thus, TBS is thought to have a distinct role in the neural network of the hippocampal slice preparation. However, the specific mechanisms that make TBS induce such neural circuit modifications are still unknown. Using electrophysiology and voltage-sensitive dye imaging (VSDI), we have found that TBS induces augmentation of spike firing. The augmentation was apparent in the first couple of brief burst stimulation (100 Hz four pulses) on a TBS-train in a presence of NMDA receptor blocker (APV 50 μM). In this study, we focused on the characterizes of the NMDA independent augmentation caused by a pair of the brief burst stimulation (the first pair of the TBS; paired burst stimulation-PBS). We found that PBS enhanced membrane potential responses on VSDI signal and intracellular recordings while it was absent in the current recording under whole-cell clamp condition. The enhancement of the response accompanied the augmentation of excitatory postsynaptic potential (EPSP) to spike firing (E-S) coupling. The paired burst facilitation (PBF) reached a plateau when the number of the first burst stimulation (priming burst) exceeds three. The interval between the bursts of 150 ms resulted in the maximum PBF. Gabazine (a GABA_A_ receptor antagonist) abolished PBF. The threshold for spike generation of the postsynaptic cells measured with a current injection to cells was not lowered by the priming burst of PBS. These results indicate that PBS activates the GABAergic system to cause short-term E-S augmentation without raising postsynaptic excitability. We propose that a GABAergic system of area CA1 of the hippocampus produce the short-term E-S plasticity that could cause exaggerated spike-firing upon a theta-gamma activity distinctively, thus making the neural circuit of the CA1 act as a specific amplifier of the oscillation signal.

## Introduction

Oscillatory neuronal activity plays pivotal roles in brain function (Buzsáki, 2006; Paulsen and Sejnowski, 2006; Sejnowski and Paulsen, 2006; Bartos et al., 2007; Fries et al., 2007). The concomitant occurrence of high- and low- frequency oscillations in many regions of the brain might work in concert to provide information to the neural network (Buzsáki and da Silva, 2012). Oscillations are a means for constructing spike firing patterns that encode information underlying distinct neuronal functions (Singer and Gray, 1995; Tiesinga et al., 2008). In the hippocampus, theta oscillations (4–8 Hz), such observed in animal exploration and learning (O’Keefe and Dostrovsky, 1971), occur together with gamma oscillations (25–100 Hz) to enable gamma-theta coding that plays a critical role (Buzsáki, 2002, 2005). Indeed, theta oscillations accompany spike generation in pyramidal cells (Ranck, 1973); the timing of which corresponds to basal oscillations critical for information encoding (O’Keefe and Recce, 1993).

An *in vitro* model of patterned stimulation that mimics theta oscillations (theta burst stimulation (TBS); TBS (a train of brief 100 Hz burst stimulations repeated in 5–7 Hz)) was found to induce long-term synaptic potentiation (LTP) at excitatory synapses (Larson and Lynch, 1986; Larson et al., 1986; Huerta and Lisman, 1995; Larson and Munkácsy, 2015). LTP induced by TBS is different from that caused by a “tetanic” high-frequency stimulus (HFS) in that these two forms of LTP exhibit different sensitivities to regulatory factors such as BDNF (Korte et al., 1995, 1996; Kang et al., 1997; Chen et al., 1999; Edelmann et al., 2015) and Ab42 (Smith et al., 2009). The mechanism of the difference is still not fully understood (Larson and Munkácsy, 2015). We have found differences in neuronal responses to these patterned stimuli during the induction stimulation of LTP. A 100 Hz HFS inhibited spike generation during the period of stimulation, on the contrary, TBS augmented spikes during the period of TBS train from the first pair of the brief burst stimulation (Tominaga et al., 2002).

These short-term modifications of synaptic responses occurred in the presence of NMDA-receptor inhibitor (APV 50 μM) and were reproducible within a short interval with any long-term modification. We showed that the short-term plasticity caused by HFS was dependent on GABA_A_ receptor (Tominaga and Tominaga, 2010). The augmentation of spike firing during TBS can be caused by the modification excitatory postsynaptic potential (EPSP) to spike generation (excitation-spike, E-S) coupling similar to that caused in parallel to the LTP induction (E-S potentiation; Andersen et al., 1980; Abraham et al., 1987; Chavez-Noriega et al., 1989, 1990; reviewed by Daoudal and Debanne, 2003). While the latter is a long-term plasticity, comparison of the controlling mechanisms of spike generation should be important. The ability of TBS to control action potential firing likely plays a critical role in controlling plasticity, as firing properties have great importance in plasticity by timing action potentials and controlling synaptic strength (Markram et al., 1997; Bi and Poo, 1998; Song et al., 2000).

On the other hand, since the short-term augmentation by TBS was apparent at the first pair of the burst stimulation, similarity to the well-known short-term plasticity of the paired pulse facilitation (PPF; Creager et al., 1980; McNaughton, 1980, 1982) should also be pursuit. It is also interesting to seek the short-term plasticity mechanism that can explain the longer range of time window than 30–60 ms of PPF applicable to theta range of events.

We recently found that high-frequency stimulation (100 Hz) induced GABA_A_-receptor-dependent long-lasting depolarization, which in turn inhibits excitatory neural signal propagation around the stimulating electrode (Tominaga and Tominaga, 2010) during HFS. Because TBS consists of the same 100 Hz burst while it is very brief (only 4–5 pulses), it is possible that same GABAergic modification caused TBS-induced short-term plasticity through the short-term plastic change of the GABAergic system (reviewed by Kaila et al., 2014a,b).

We, therefore, aimed to explore the neuronal mechanisms by which TBS regulates spike firing in a simple single input *in vitro* model of gamma-theta interactions. We further tested for the possible involvement of GABA_A_-receptor-mediated action potential firing control, which would represent a network driven mechanism of TBS regulation of neuronal activity.

## Materials and Methods

### Slice Preparation and Staining with VSD

All animal experiments were performed according to protocols approved by the Animal Care and Use Committee of Tokushima Bunri University. Hippocampal slices were prepared from 4-5-week-old male Wistar rats that were decapitated under deep isoflurane anesthesia after perfusion with ice-cold artificial cerebrospinal fluid (aCSF; 124 mM NaCl, 2.5 mM KCl, 2 mM CaCl_2_, 2 mM MgSO_4_, 1.25 mM NaH_2_PO_4_, 26 mM NaHCO_3_, and 10 mM glucose; pH 7.4) bubbled with 95%/5% O_2_/CO_2_ gas. The brains were quickly removed and cooled in aCSF. After cooling for 5 min, the hippocampus and surrounding cortex were dissected and sliced into 400 μm transverse sections using a vibratome (Leica VT-1000 or VT-1200S). Following incubation in gassed aCSF for 3–5 min, each slice was transferred onto a fine-mesh membrane filter (Omni Pore membrane filter, JHWP01300; Millipore Corp., MA, USA) held in place by a thin Plexiglas ring (inner diameter, 11 mm; outer diameter, 15 mm; thickness 1–2 mm). These slices were transferred to a moist chamber continuously supplied with a humidified O_2_/CO_2_ gas mixture. The temperature was held at 32°C for 1 h, and then maintained at room temperature thereafter.

After 1 h of incubation, each slice was stained for 25 min with 100 μl of voltage sensitive dye (VSD) solution containing 0.2 mM Di-4-ANEPPS (Molecular Probes) in 2.5% ethanol, 0.13% Cremophor EL (Sigma), 1.17% distilled water, 48.1% fetal bovine serum (Sigma), and 48.1% ACSF. After washing to remove VSD, sections were incubated at room temperature for least 1 h before they were imaged by optical recording.

### Optical Recording

The Plexiglas ring supporting each slice was placed in an immersion-type recording chamber. Slices were continuously perfused with pre-warmed (31°C) and oxygenated aCSF (bubbled with a 95%/5% O_2_/CO_2_ gas mixture) at a rate of 1 ml/min. Custom laboratory-designed epifluorescence optics consisting of two principal lenses were used to view the slices during experiments. The optics consisted of a custom-made objective lens (Olympus MYCAM 5×/0.6 WI; the final magnification of the system was 5×) as the objective lens, and a (*f* = 55 mm × 1.0) Leica Microsystems MZ-APO lens as the projection lens. Excitation light was provided by a halogen lamp source (150 W; MHW-G150LR; Moritex Corp.) and was projected through an excitation filter (*λ* = 530 ± 10 nm) and reflected onto the hippocampal slice by a dichroic mirror (*λ* = 575 nm). Emission fluorescence from the slice was passed through an emission filter (*λ* > 590 nm) and projected onto a CCD camera (MiCAM01 and MiCAM Ultima; BrainVision, Inc., Tokyo, Japan). The high-speed imaging system provided a spatial resolution of approximately 22 × 22 μm at the objective (96 pixels × 64 pixels resolution; MiCAM01) and 18.2 × 18.2 μm (100 pixels × 100 pixels resolution; MiCAM Ultima).

The intensity of emitted slice fluorescence prior to stimulation (a pre-stimulation period usually lasted 40 frames) was averaged and used as a reference intensity (*F*_0_). The fractional change in fluorescence [Δ*F*_(t)_ = *F*_(t)_−*F*_0_] was normalized by *F*_0_ (Δ*F*/*F*_0_), and this value was used as the optical signal. Optical signals referred to in the following sections represent signals filtered in spatial and temporal dimensions with a Gaussian kernel of 5 × 5 × 3 (horizontal × vertical × temporal).

We analyzed optical signals offline using a procedure developed for IgorPro (WaveMetrics Inc., OR, USA). At a wavelength of 610 nm, VSD fluorescence decreases in response to the depolarization of the membrane. To fit the polarity of the response to conventional membrane potential changes, we expressed the optical signal in a polarity that matched the membrane potential change. For example, decreased fluorescence, which corresponds to depolarization, was represented as a positive deflection. For additional details on the methods, see Tominaga et al. (2000, 2002).

### Electrophysiological Recording and Stimulation

Patch-clamp recordings in whole-cell mode were obtained using a patch-clamp amplifier with a capacitive headstage (Axoclamp 700B; Axon Instruments, Foster City, CA, USA). Pipettes of borosilicate glass (Sutter Instruments, Novato, CA, USA) were pulled using a P-97 Flaming-Brown pipette puller (Sutter Instruments, Novato, CA, USA). The Cs-based pipette internal solution used for whole-cell voltage clamp experiments consisted of (in mM): 130 Cs-MeSO_3_, 10 HEPES, 4 MgCl_2_, 4 NaATP, 0.4 NaGTP, 10 Na-phosphocreatine, 10 EGTA; pH was adjusted to 7.2; 5 mM QX-314 was also added (2–4 MΩ).

Whole-cell recordings were low-pass-filtered at 3 kHz and digitized at 10 kHz (ITC-18; InstruTech Inc., NY, USA). Data were fed into an Apple computer for on-line and off-line analysis using laboratory-developed software on IgorPro (WaveMetrics Inc., OR, USA). Electrical stimuli were constant current pulses (A395, WPI) applied through a glass microcapillary pipette (5 μm inner diameter; filled with aCSF). Neurons were visualized by oblique illumination with the aid of contrast enhancement provided by a CMOS camera (SKDCE-2EX; Sigma Koki Co., Tokyo, Japan) attached to an upright microscope (BX-51WI; Olympus Tokyo, Japan). In voltage-clamp mode, a test membrane potential step (−10 mV) was always applied prior to electrical stimulation, and traces with series resistance (Rs) lower than 25 MΩ were accepted. Membrane current at the holding potential of −70 mV were presented.

For intracellular recording, we used a fine-tipped glass microelectrode filled with 4 M potassium acetate (approximately 100 MΩ). An Axoclamp-2B amplifier (Axon Instruments) was used in continuous bridge mode. Cells with resting potentials of −65 to −80 mV were accepted for study.

Stimulation electrode was placed in the middle of the stratum radiatum (about 200 μm from the startum pyramidale) at the border between the CA1 and CA3. Whole cell recordings and intracellular recordings were applied to the cells in the middle of CA1 (about 500 μm from the stimulating electrode).

The electrophysiological recording system was controlled by a procedure developed in Igor Pro (WaveMetrics Inc., OR, USA). Artifacts caused by electrical stimulation were digitally removed from the traces shown in the Results. To monitor synaptic transmission in CA1 we applied a 0.05–0.1 Hz stimulus at an intensity that produced an approximately 30% maximal field excitatory postsynaptic potential (fEPSP).The fEPSPs were always monitored with a field potential electrodes placed at the most distal end of the area CA1 in all the experiments. A laboratory-made differential amplifier and a general-purpose amplifier (model 440; Brownlee Precision, San Jose, CA, USA) were used for field potential recordings. A glass microcapillary pipette (5 μm inner diameter; filled with aCSF) was used as a recording electrode for field potential recordings. The electrophysiological recording and the optical recording did not interfere with each other.

### Drugs and Solutions

All experiments were conducted in the presence of 50 μM DL-2-amino-5-phosphonovaleric acid (APV, Tocris), unless otherwise stated. We took advantage of the absence of synaptic plasticity effects in the presence of APV to apply multiple episodes of tetanic stimulation in a single slice and to improve the signal-to-noise (S/N) ratio of optical signals by averaging. SR95531 (gabazine) was dissolved in water to make a 10 mM stock solution. Other common reagents were obtained through local resellers in Japan.

### Data Analysis

Optical and electrophysiological signals were analyzed using custom macro programs on Igor Pro (WaveMetrics Inc., OR, USA). All data are expressed as the mean ± SEM; and *n* represents the number of slices. Statistical significance was tested with Tukey’s mutiple comparisons after one-way analysis of variance (ANOVA) using Igor Pro (WaveMetrics Inc., OR, USA).

## Results

### Paired Burst Transiently Increased Postsynaptic Membrane Potential Responses in CA1 Pyramidal Cells upon Schaffer Collateral Stimulation

**Figure 1:**
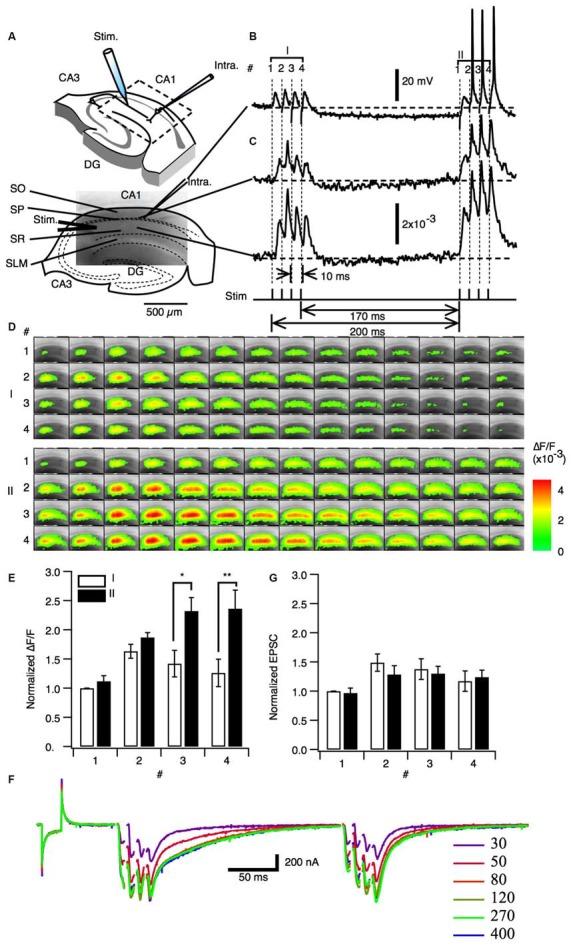
**Paired priming burst stimulation increased excitatory postsynaptic transmission in later burst stimulations. (A)** Upper image: Schematic illustration of a hippocampal slice with a stimulation electrode (Stim). A field of view of the optical recording is shown in the image embedded in the square inset. The image in the lower panels shows a fluorescent image (ca. 2 mm × 1.3 mm) of the area of CA1 imaged by optical recording. Abbreviations: SO, stratum oriens-alveus; SP, stratum pyramidale; SR, stratum radiatum; SLM, stratum lacunosum-moleculare. **(B)** Representative intracellular membrane potential trace recorded with a sharp microelectrode. **(C)** Representative optical signal of a pixel acquired from the stratum pyramidale (upper panel) and a pixel acquired from the middle of the stratum radiatum (lower panel). Protocol of the paired burst stimulation are shown in the bottom of **(C)**. The stimulation consisted of the priming burst stimulation (I: 100 Hz 4 pulses) and the test burst stimulation (II: 100 Hz 4 pulses) with 170 ms interval. **(D)** Sets of consecutive images of optical signals sampled every 0.7 ms for the priming burst stimulation (I) and the test burst stimulation burst (II) of stimulation numbers 1–4 (top to bottom). **(E)** Amplitude of the optical response upon test simulation (I; open bars) and the following stimulation burst (II; filled bars; mean ± SEM; *p < 0.05, **p < 0.01; *n* = 6). **(F)** Membrane current response under whole-cell clamp conditions (holding potential = −70 mV) at different stimulus intensities. **(G)** Amplitude of the EPSCs recorded for the burst stimuli (I) and (II). (mean ± SEM; *n* = 5).

We first sought to understand the effect of neural activity similar to theta-bursts on facilitation in the hippocampus. A set of four pulses of burst stimulation (100 Hz) to the Schaffer collateral pathway with subthreshold stimulus intensity did not elicit spikes under sharp electrode intracellular recording (Figure 1B stimulation I). However the same set of four pulses of burst stimulation at same stimulus intensity after 170 ms (stimulation II) elicited spike firing (Figure 1B stimulation II). Hereafter we used this paired burst stimulation (PBS) pattern [a 100 Hz burst stimulation consists of four stimuli with10 ms interval (a priming burst; I) proceeds 170 ms of interburst interval to the same 100 Hz burst stimulation (a test burst; II)] illustrated in the Figures 1B,C as a PBS unless otherwise stated. The PBS of 170 ms of interburst interval is identical to the first pair of the TBS. This result is consistent with a finding that a train of burst stimuli that mimics theta-burst TBS showed progressive facilitation of action potential generation (Tominaga et al., 2002). Please note that the response to the PBS and monitoring fEPSPs to single stimulation were consistent at least when the PBS applied every 20 s, so that the facilitation did not last more than 20 s. We next examined the response to the same PBS paradigm throughout the slice by analyzing optical signals using a VSD imaging method. We observed that augmentation of the postsynaptic responses occurred in both the stratum pyramidale (SP) (Figure 1C top trace) and stratum radiatum (SR) (Figure 1C bottom trace) across most of CA1 (Figure 1D). We evaluated the amplitude of the response to each stimulus (1–4) of the priming burst stimulus (I in the Figures 1B–E) and the following test burst stimulus (II) by measuring peak amplitude of the response in the middle of the stratum radiatum. As shown in Figure 1E, the responses to the test burst (filled bars) were significantly larger than those to the priming burst (open bar) in the 3rd and 4th stimulus. Since a pair of 100 Hz burst stimulation caused transient facilitation of the response to the subsequent burst stimulation, we refer this short-term plastic change in postsynaptic response as a paired burst facilitation (PBF).

We next asked whether the PBF is caused by the modification of the synaptic transduction by whole cell clamp experiments. Excitatory postsynaptic current (EPSC) were measured with whole cell clamp condition with the internal solution adjusted to minimize inhibitory current at the holding potential of −70 mV (E_Cl_ = −70 mV). There were no significant difference between the postsynaptic responses in the priming burst (I) and test burst (II) under voltage clamp conditions (Figures 1F,G). Therefore, the PBF was specific to membrane potential responses and not due to increased synaptic receptor currents.

### PBF Accompanied Transient Augmentation of Excitation-Spike (E-S) Coupling

**Figure 2:**
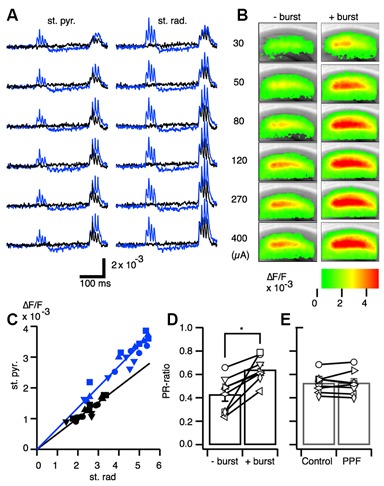
**Excitation-spike generation potentiation caused by paired burst facilitation. (A)** Representative traces of optical signals acquired from the stratum pyramidale (st. pyr.) and stratum radiatum (st. rad.) at stimulus intensities of 30, 50, 80, 120, 270, and 400 μA with (blue) and without (black) a priming burst stimulation. **(B)** Pseudo-colored image of the projection of the maximum amplitude of the response to the burst stimulation without a priming burst (−burst) and with a priming burst (+burst) at each of the stimulus intensities. **(C)** Representative relationship of the optical signal amplitude in the stratum pyramidale and stratum radiatum without a priming burst (black) and with a priming burst (blue). **(D)** Ratio of the response in the stratum pyramidale and stratum radiatum (PR-ratio; *p < 0.01; *n* = 8). **(E)** The same analysis as in **(D)** conducted for the paired-pulse facilitation protocol (inter-stimulus interval = 40 ms); no significant differences were found between the single stimulus and paired stimulus conditions.

Given that TBS has been shown to increase the chance of spike firing (Tominaga et al., 2002), we examined the relationship between synaptic potential and spike-firing during PBF. Facilitation in the magnitude of the optical responses in CA1 was observed in response to a range of stimulus intensities (Figures 2A,B).

Plotting the relationships between the response in the SR and SP in the control condition (without priming burst; black symbols) and in the test condition (with priming burst; blue symbols) revealed a linear relationship in both regions (Figure 2C). Moreover, the slopes were higher with a priming burst than without priming.

The optical signal in the SR is dependent on EPSP, while in the SP is dependent on the spike occurrence (Tominaga et al., 2009). Thus, the larger slope elicited with the PBS condition implies there was an enhanced excitation-spike (E-S) firing relationship (i.e., augmentation of E-S coupling). Therefore, we here-to-fore use the ratio of the response of the SP and that of the SR (PR-ratio) as a measure of the strength of the E-S relationship (Tominaga et al., 2009). The PR-ratio in the test condition (+ burst) was significantly larger than the control (−burst; Figure 2D). That is, PBF accompanied transient short-term modification of E-S coupling. By contrast, the same relationships measured using the well-established short-term plasticity caused by the PPF protocol (Creager et al., 1980; McNaughton, 1980, 1982) did not exhibit changes in the PR-ratio (Figure 2E). Thus, the PBF might require different mechanisms than well-established forms of PPF caused by the presynaptic mechanisms (Manabe et al., 1993; Manabe and Nicoll, 1994).

### Effect of the Number of the Priming Burst and Interburst Interval of PBS

**Figure 3:**
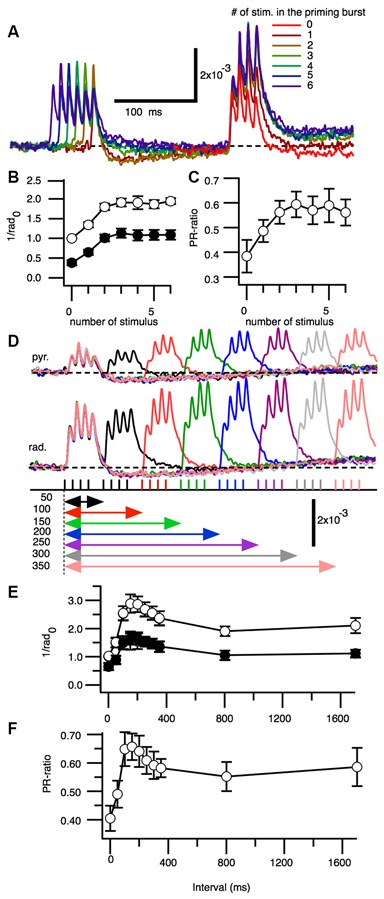
**Effect of increasing numbers of burst stimulations and burst repetition intervals on the facilitation of excitatory synaptic transmission. (A)** Superimposed representative traces of the optical signals acquired from the stratum radiatum after zero to six burst stimulations. The amplitude of the third peak of the response was measured. **(B)** Pooled data for the amplitude of the test responses normalized by the control response (no priming burst stimulation) at the stratum radiatum (1/rad_0_) in the stratum pyramidale and stratum radiatum, as a function of the number of priming bursts. **(C)** Ratio of the stratum pyramidale and stratum radiatum amplitudes as a function of the priming burst (*n* = 6). **(D)** Representative set of optical signals acquired from the stratum pyramidale (st. pyr.) and stratum radiatum (st. rad.) at different burst intervals (50–350 ms). **(E)** Change in the normalized maximum amplitude [normalized by the control response (no priming burst stimulation) at the stratum radiatum (1/rad_0_) of the test burst stimulation as a function of burst intervals. **(F)** Ratio of the response between the stratum pyramidale and stratum radiatum (PR-ratio) as a function of burst intervals (*n* = 5; Bars indicate the SEM).

We next assessed if PBF caused by “burst” stimuli affected facilitation and found that optical signals increased to their maximum values with either three or four priming burst stimulations (one to six stimuli were tested; Figure 3A). The maximum amplitude was observed in response to the third stimulation in SR and SP. Facilitation was highest when there were four stimulations. The PR-ratio increased as the number of the priming burst stimulations increased, until reaching the peak value at four stimulations (Figure 3C).

We next tested the optical signals elicited by PBS at different interburst intervals (Figure 3D). The amplitude of the response and E-S potentiation reflected in the PR-ratio were greatest when the interval was approximately150 ms (Figures 3E,F).

### PBF is Dependent on GABA_A_-Receptor Activation

**Figure 4:**
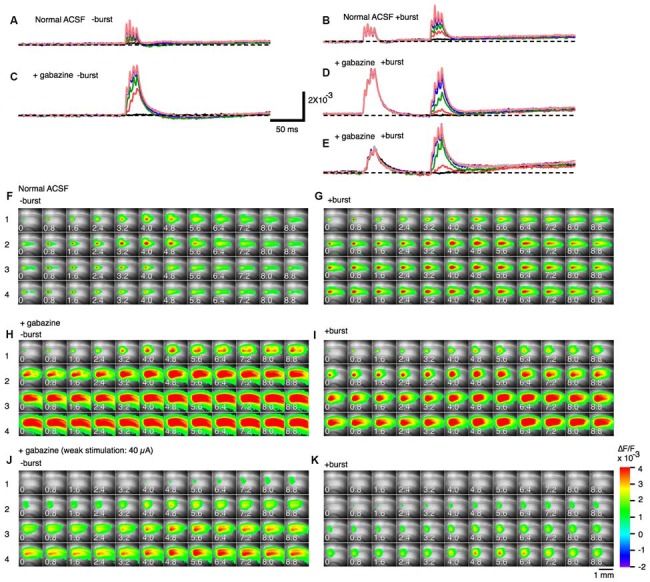
**Effect of a GABA_A_ receptor antagonist (gabazine) on paired burst facilitation. (A–D)** Representative traces of optical signals observed upon burst stimulation at different stimulus intensities with (**A,C**; +burst) or without (**B,D**; −burst) a priming burst stimulation (100 μA) in normal ACSF **(A,B)** and in the presence of 10 μM gabazine **(C,D)**. **(E)** The stimulus intensity of a priming burst stimulation was reduced to 40 μA. The test burst stimulation were 20, 40, 60, 100, 150, 200 and 250 μA. **(F–K)** Sets of consecutive images of optical signals sampled every 0.8 ms for the priming burst stimulation **(F,H,J)** and the test burst stimulation burst **(G,I,K)** of stimulation numbers 1–4 (top to bottom). The stimulation intensity of both priming and test burst stimulation were 100 μA in **(F–I)**, while were 40 μA in **(J,K)**. The response **(H–K)** are taken in the presence of 10 μM gabazine. Small numbers on each consecutive images are time from the starting of each stimulation of a brief burst stimulation (1–4) (ms).

A 100 Hz stimulation is known to cause reversal of GABA_A_-receptor currents from hyperpolarizing to depolarizing membrane potential responses (Buzsáki et al., 2007; Kaila et al., 2014a) and cause several short-term plasticity in area CA1 (Tominaga and Tominaga, 2010). Thus, PBS might be achieved through a GABA_A_-receptor-mediated mechanism. We tested this by applying GABA_A_ a receptor antagonist to the hippocampal slice media and assessing neural activity (SR95531 or gabazine, 10 μM; Figure 4). Application of gabazine could cause saturation of the response so that we tested responses in normal ACSF and inhibitor-treated ACSF at different stimulus intensities with or without priming burst stimulation (+burst vs. –burst; Figures 4A,B; stimulation intensity of the priming burst was 100 μA). That is clear when comparing response pattern with and without priming burst stimulation at both stimulation intensity was same (100 μA; Figures 4F,G). Application of gabazine enhanced the response in the control condition (−burst; Figures 4C,G), but burst stimulation failed to induce facilitation in the presence of GABA_A_ receptor inhibitors (Figures 4D,I; priming burst 100 μA). That is also true if the stimulation intensity of the priming burst was lowered to 40 μA (Figures 4E,J,K) where the priming burst stimulation did not saturate the response.

**Figure 5:**
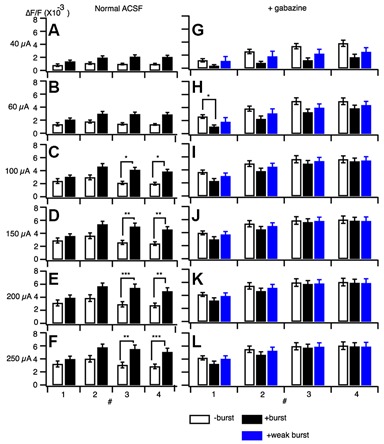
**Amplitude of responses at different stimulus intensities without a burst stimulation (open bars) and with a priming burst stimulation (filled bars) in normal ACSF (A–F) and in the presence of gabazine (G–L).** The stimulation intensity of a priming burst was 100 μA for black bars, and 40 μA for blue bars in **(G–L)**. (mean ± SEM; *p < 0.05, **p < 0.01, ***p < 0.001; *n* = 7).

That was clearer in the pooled data (Figure 5). The differences caused by the priming burst stimulation were significantly larger at stimulus intensities greater than 100 μA in the stimulus #3 and #4 (Figures 5D–F). In presence of gabazine, there were no significant increase in the response caused by the priming burst stimulation (compare open and black filled bars in Figures 5G–L). Again that is not due to the saturation effect caused by gabazine, because even in the small stimulus intensity (40 μA) failed to cause the facilitation (compare open and blue filled bars in Figures 5G–L), strongly indicating that activation of GABA_A_-receptors is a critical factor in PBF. It is interesting to note that in presence of gabazine priming burst tend to reduce the following response (significant decrease only seen in Figure 5H #1 stimilus open and black bars).

### Transient E-S Augumentation Without a Decrease in Spike Generation Threshold

**Figure 6:**
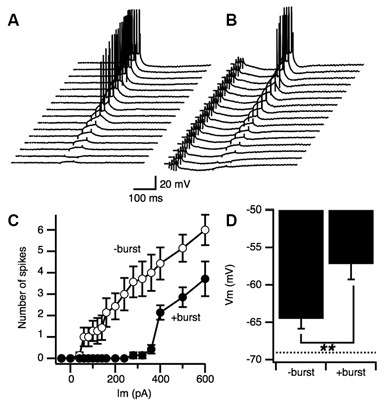
**Neuronal excitability was not increased by a priming burst stimulation. (A)** Set of traces of intracellular recordings with a sharp intracellular electrode at different amplitudes elicited by a square constant current injection. **(B)** Matching series of traces recorded with a priming synaptic burst stimulation before the constant current injection. **(C)** Number of spikes produced during the current injection without burst stimulation (open circle) and with burst stimulation (filled circle) as a function of the amplitude of the current. Bars represents SEM (*n* = 6 for both groups). **(D)** Membrane potential of the first appearance of spikes when the intensity of injection current increased in the condition of without a priming burst stimulation (−burst) and with the priming burst stimulation (+burst). Dashed line represents the resting potential (−69.06 ± 1.43; *n* = 12). **p < 0.01.

We next examined if the E-S augumenation induced by the priming burst stimulation accompanied modifications of spike generation threshold in postsynaptic cells. The threshold for spike generation was examined by directly injecting current into pyramidal cells through a fine tipped intracellular electrode with or without priming synaptic burst stimulation (Figures 6A,B). When the priming burst stimulation was given 170 ms before the current injection like in the case of PBS, number of the spikes caused by the injected current were suppressed (Figure 6C open circle) compared to those without priming burst (Figure 6C close circle). The membrane potential that induce the first action potential was significantly lower in the control than with priming burst (Figure 6D) That led us to confirm an increase in spike generation threshold (Figure 6C), indicating that greater postsynaptic excitability is not the cause of PBS induced E-S augmentation.

## Discussion

In the present study, we showed that a pair of 100 Hz brief burst stimulations (PBS) to the Schaffer collateral afferent induced a transient facilitation of the postsynaptic response (PBF) in the intracellular recordings and VSD optical recordings in the area CA1 of rat hippocampal slices in the presence of an NMDA blocker. Since the same PBS did not cause the facilitation of EPSC under a voltage-clamp condition, PBF was not caused by the change in presynaptic nor the postsynaptic-receptor modification. The PBF accompanied the increase in the EPSP-spike (E-S) coupling. This short-term modification of E-S coupling was seen when the priming burst of the PBS consisted of more than three and the interburst frequency was 150 ms. The optimal stimulation pattern was close to the TBS that originally aimed to mimic the natural oscillatory activity of the hippocampus (Larson and Lynch, 1986; Larson et al., 1986). The PBF was abolished when the GABAergic inhibitory circuit was blocked by the application of gabazine and did not accompany the lowering of the spike threshold of the postsynaptic cells. Taken together the PBF is a short-term plastic facilitation of excitation and E-S coupling of the postsynaptic pyramidal cells of area CA1 caused by transient modification of GABAergic systems. These results imply that CA1 neural circuit equips the theta-dependent amplifier that specifically amplified spike generation to the theta-gamma combined activity. In other words, the PBF could be a part of the key mechanism that enable CA1 to decode the theta oscillatory neural signal as a meaningful signal.

### Comparison with the Paired-Pulse Facilitation (PPF) and the E-S Potentiation

The PBF is a short-term activity modification as is the representative short-term plasticity PPF that occurs when a pair of a stimuli were given to same afferent in rapid (intervals less than 100–200 ms) succession (Creager et al., 1980; McNaughton, 1980, 1982). In the case of the PPF, it is well established that the presynaptic mechanism contributes to the causing it (Manabe et al., 1993; Schulz et al., 1995; Debanne et al., 1996). Although PPF involves GABAergic contribution (Davies et al., 1990, 1991), PBF should be different from PPF in at least two aspects. Firstly the range of the time-window is longer than that in PPF. PBF has a peak at 150 ms (Figure 3) while PPF is at 30–60 ms (Creager et al., 1980; McNaughton, 1980, 1982). Secondly, PPF could be observed in the EPSC (Manabe et al., 1993) but PBF could not (Figure 1). Hence, the PBF and the PPF are both short-term modifications of neuronal response but should be caused by different mechanisms, at least, in part.

The PBF accompanied a transient increase in E-S coupling. A long-term modification of E-S coupling is well known as an E-S potentiation that is complementary to the induction of LTP (Andersen et al., 1980; Abraham et al., 1987; Chavez-Noriega et al., 1990) and was already noted on the first report of LTP (Bliss and Lømo, 1973). The E-S potentiation (and E-S depression) is a long-term modification of E-S relationship induced by tetanic stimulation and other forms of stimulation (Pugliese et al., 1994; review by Daoudal and Debanne, 2003). Both the modification of intrinsic neuronal excitability of postsynaptic neurons and the modification, including the GABAergic system are responsible for the E-S potentiation and E-S depression depending on the induction condition of LTP (Chavez-Noriega et al., 1989, 1990; Hess and Gustafsson, 1990; Jester et al., 1995; Lu et al., 2000; Daoudal et al., 2002; Marder and Buonomano, 2003; Staff and Spruston, 2003). While PBF did not accompany a lowering of spike threshold (Figure 6), GABAergic mechanisms that cause the long-term modification of neural response that resulted in E-S potentiation can apply to the cause of PBF (see Figures 4, 5). However, thinking about a large difference between short-term and long-term neural modification, the way of involvements of these mechanisms should be different.

### How Does GABA_A_-Receptor-Mediated Network activity Cause Paired Burst Facilitation (PBF)

GABAergic inhibition operates by a variety of mechanisms including hyperpolarization and shunting of postsynaptic cells (Bartos et al., 2007; Mann and Paulsen, 2007; Blaesse et al., 2009). The GABA_A_-receptor-mediated inhibitory system consists of two kinetically different currents GABA_A, fast_ and GABA_A, slow_ (Pearce, 1993), in diverse types of interneurons (reviewed by Maccaferri and Lacaille, 2003). The time constant for GABA_A, fast_ is around 9 ms and GABA_A, slow_ is around 50 ms (Banks et al., 1998). Those kinetically different GABA_A_-receptors of different types of interneurons are thought to be essential to support nested activity of theta and gamma frequencies (Banks et al., 2000; White et al., 2000; Bartos et al., 2002, 2007). The optimal interburst interval of 150 ms in the present data may imply other processes that should explain a little bit longer time constants than GABA_A, slow_. In this connection, it is interesting to note that high-frequency stimulation causes long-lasting depolarization instead of hyperpolarization (Kaila et al., 2014b). This form of depolarization is considered to result from a depolarizing response to excess administration of GABA (Nicoll and Alger, 1979) that also induces tonic activation of extrasynaptic GABA_A_ receptors (Semyanov et al., 2004). Accumulating evidence indicates that the GABA_A_-receptor mediated depolarizations are closely related to the equilibrium potential of the Cl^−^ (E_Cl_) shift, which depends on the activity of cation-chloride cotransporters (Buzsáki et al., 2007; Fiumelli and Woodin, 2007; Blaesse et al., 2009; Kaila et al., 2014b). The shift in ECl caused so called “ionic plasticity” that could provide longer processes than each receptor current (Kaila et al., 2014b). We have previously shown that high-frequency stimulation at 100 Hz caused long-lasting GABA_A_ receptor-dependent depolarization leading to the inhibition of synaptic transmission followed by a facilitation of neuronal activity transduction (Tominaga et al., 2002; Tominaga and Tominaga, 2010). The short-term plasticity caused by 100 Hz stimulation (duration of 400 ms) occurred in less than a second. Hence, we propose that a brief 100 Hz burst stimulation of TBS and PBS may recruit a process that is causing ionic plasticity. In the present study, we found that long-lasting GABA_A_ receptor-dependent depolarization induced by PBS did not simply increase the depolarizing response, as indicated by the heightened threshold for spike generation in response to a current injection (Figure 6). In addition, there was no evidence for presynaptic modulation by GABA_A_-receptors (Wakita et al., 2014), as there was no facilitation of EPSCs (Figure 1). It is still difficult to explain the raised E-S coupling from these controls of inhibitory systems. However, based on the present results and given that perisomatic feed-forward inhibition by GABA_A_ receptors controls action potential firing (Freund and Katona, 2007; Tominaga et al., 2009), temporal weakening of feed forward inhibition, i.e., dis-inhibition of feed-forward inhibition, seems a part of an explanation for the present results (Lu et al., 2000).

### PBF as an Intrinsic Neural Mechanism Generated by Gamma-Theta Oscillations

The 100 Hz burst stimulation protocol used in the present study can be considered to be a form of high-frequency oscillations (HFO; Engel and da Silva, 2012) in the gamma range. In various brain regions, HFO is often observed together with low-frequency oscillations, such as theta oscillations (Buzsáki and da Silva, 2012). In the hippocampus, gamma-theta interactions are thought to be a crucial computational mechanism for processing neural information (Lisman and Jensen, 2013; Nishida et al., 2014). Theta oscillations have long been known to play an important role in encoding information in neural circuits (Buzsáki, 2005) and in memory consolidation during sleep (Mehta et al., 2002). Control of these spikes in entorhinal-hippocampal connections during slow oscillatory activity is critical for hippocampal function (Ahmed and Mehta, 2009). For instance, projections from the medial entorhinal cortex (MEC) layer III mediate ripple activity in the CA1 (Suh et al., 2011) and control memory processes in the hippocampus (Yamamoto et al., 2014). Moreover, the timing of spikes during theta oscillations is crucial for computation in the hippocampus, as most notably indicated by the phase precession of spikes related to place cell tuning (O’Keefe and Recce, 1993).

The *in vitro* data presented in this study suggest that the CA1 network has an intrinsic mechanism to control spike firing upon the coherent activation of Schaffer collaterals by theta oscillations. This may be mediated at least in part by dendritic K^+^ channels that act as cellular devices that regulate the theta frequency time domain (Watanabe et al., 2002). Our results show that the GABA_A_-receptor activation induced by bursting synaptic input may additionally regulate neural circuitry within CA1 by enabling more efficient transmission of theta range neural transduction. Spike firing in CA1 pyramidal cells is strictly controlled by neuronal inhibition, mostly via perisomatic GABAergic synapses which are abundant in this region (Thompson and Best, 1989; Tominaga et al., 2009). It however remains to be seen how perisomatic inhibition exerts precise control over spike firing in individual cells. A reasonable explanation for the cellular basis for GABA-dependent spike firing regulation may be intrinsic mechanisms that respond to the oscillatory activity of inputs into the CA1.

### LTP Induction by Different Stimuli

The present study confirmed that the TBS induces more spikes during the induction of LTP, than the number of spikes that are inhibited by HFS (Tominaga et al., 2002). In consideration of the classical definition of Hebbian plasticity (Hebb, 1949; Stent, 1973), the physiological importance of spikes for the induction of LTP is quite important. In addition, differences in the forms of LTP (Larson and Munkácsy, 2015) elicited by TBS vs. HFS may owe to different sets of molecular cascades initiated by these two distinct processes (Zhu et al., 2015) or it could be caused by differences in the nature of spikes coincident with the synaptic input (Edelmann et al., 2015). We found that spike generation by TBS was controlled by the GABA system, indicating a potential coupling of interneurons and Schaffer collateral inputs onto postsynaptic cells in the circuit (Buzsáki et al., 2004, 2007). The involvement of inhibitory pathways may be important in establishing variability in the ways in which the network is differentially modified by various LTP induction stimuli. Given that LTP induction is still used to test risk for and dysfunction in many brain disorders and malformations (e.g., risk assessment of chemical factors and genetic backgrounds in Alzheimer’s disease), it is important to consider that processes underlying LTP induction may differ according to the type of stimulus.

## Author Contributions

TT designed research; TT and YT performed research; TT and YT analyzed data; TT wrote the article.

## Funding

Grant from Ministry of Health, Labour and Welfare (H23-Kagaku-Ippan-004, H27-Kagaku-Ippan-007), JST A-STEP, KAKENHI 24500269, 24240076, 15K00413 to TT.

## Conflict of Interest Statement

The authors declare that the research was conducted in the absence of any commercial or financial relationships that could be construed as a potential conflict of interest.

## References

[B1] Abraham W. C. Gustafsson B. Wigström H. (1987). Long-term potentiation involves enhanced synaptic excitation relative to synaptic inhibition in guinea-pig hippocampus. J. Physiol. 394, 367–380. 10.1113/jphysiol.1987.sp016875 3443970 PMC1191966

[B2] Ahmed O. J. Mehta M. R. (2009). The hippocampal rate code: anatomy, physiology and theory. Trends Neurosci. 32, 329–338. 10.1016/j.tins.2009.01.009 19406485 PMC3066563

[B3] Andersen P. Sundberg S. H. Sveen O. Swann J. W. Wigström H. (1980). Possible mechanisms for long-lasting potentiation of synaptic transmission in hippocampal slices from guinea-pigs. J. Physiol. 302, 463–482. 10.1113/jphysiol.1980.sp013256 7411464 PMC1282861

[B4] Banks M. I. Li T. B. Pearce R. A. (1998). The synaptic basis of GABA_A,slow_. J. Neurosci. 18, 1305–1317. 9454840 10.1523/JNEUROSCI.18-04-01305.1998 PMC6792721

[B5] Banks M. I. White J. A. Pearce R. A. (2000). Interactions between distinct GABA(A) circuits in hippocampus. Neuron 25, 449–457. 10.1016/s0896-6273(00)80907-1 10719898

[B6] Bartos M. Vida I. Frotscher M. Meyer A. Monyer H. Geiger J. R.. (2002). Fast synaptic inhibition promotes synchronized gamma oscillations in hippocampal interneuron networks. Proc. Natl. Acad. Sci. U S A 99, 13222–13227. 10.1073/pnas.192233099 12235359 PMC130614

[B7] Bartos M. Vida I. Jonas P. (2007). Synaptic mechanisms of synchronized gamma oscillations in inhibitory interneuron networks. Nat. Rev. Neurosci. 8, 45–56. 10.1038/nrn2044 17180162

[B8] Bi G. Poo M. (1998). Synaptic modifications in cultured hippocampal neurons: dependence on spike timing, synaptic strength and postsynaptic cell type. J. Neurosci. 18, 10464–10472. 9852584 10.1523/JNEUROSCI.18-24-10464.1998 PMC6793365

[B9] Blaesse P. Airaksinen M. S. Rivera C. Kaila K. (2009). Cation-chloride cotransporters and neuronal function. Neuron 61, 820–838. 10.1016/j.neuron.2009.03.003 19323993

[B10] Bliss T. V. P. Lømo T. (1973). Long-lasting potentiation of synaptic transmission in the dentate area of the anaesthetized rabbit following stimulation of the perforant path. J. Physiol. 232, 331–356. 10.1113/jphysiol.1973.sp010273 4727084 PMC1350458

[B11] Buzsáki G. (2002). Theta oscillations in the hippocampus. Neuron 33, 325–340. 10.1016/s0896-6273(02)00586-x 11832222

[B12] Buzsáki G. (2005). Theta rhythm of navigation: link between path integration and landmark navigation, episodic and semantic memory. Hippocampus 15, 827–840. 10.1002/hipo.20113 16149082

[B13] Buzsáki G. (2006). Rhythms of the Brain. Oxford; New York, NY: Oxford University Press.

[B14] Buzsáki G. da Silva F. L. (2012). High frequency oscillations in the intact brain. Prog. Neurobiol. 98, 241–249. 10.1016/j.pneurobio.2012.02.004 22449727 PMC4895831

[B15] Buzsáki G. Geisler C. Henze D. A. Wang X.-J. (2004). Interneuron diversity series: circuit complexity and axon wiring economy of cortical interneurons. Trends Neurosci. 27, 186–193. 10.1016/j.tins.2004.02.007 15046877

[B16] Buzsáki G. Kaila K. Raichle M. (2007). Inhibition and brain work. Neuron 56, 771–783. 10.1016/j.neuron.2007.11.008 18054855 PMC2266612

[B17] Chavez-Noriega L. E. Bliss T. V. Halliwell J. V. (1989). The EPSP-spike (E-S) component of long-term potentiation in the rat hippocampal slice is modulated by GABAergic but not cholinergic mechanisms. Neurosci. Lett. 104, 58–64. 10.1016/0304-3940(89)90329-7 2554222

[B18] Chavez-Noriega L. E. Halliwell J. V. Bliss T. V. (1990). A decrease in firing threshold observed after induction of the EPSP-spike (E-S) component of long-term potentiation in rat hippocampal slices. Exp. Brain Res. 79, 633–641. 10.1007/bf00229331 2340880

[B19] Chen G. Kolbeck R. Barde Y. A. Bonhoeffer T. Kossel A. (1999). Relative contribution of endogenous neurotrophins in hippocampal long-term potentiation. J. Neurosci. 19, 7983–7990. 10479698 10.1523/JNEUROSCI.19-18-07983.1999 PMC6782442

[B20] Creager R. Dunwiddie T. Lynch G. (1980). Paired-pulse and frequency facilitation in the CA1 region of the *in vitro* rat hippocampus. J. Physiol. 299, 409–424. 10.1113/jphysiol.1980.sp013133 7381775 PMC1279233

[B21] Daoudal G. Debanne D. (2003). Long-term plasticity of intrinsic excitability: learning rules and mechanisms. Learn. Mem. 10, 456–465. 10.1101/lm.64103 14657257

[B22] Daoudal G. Hanada Y. Debanne D. (2002). Bidirectional plasticity of excitatory postsynaptic potential (EPSP)-spike coupling in CA1 hippocampal pyramidal neurons. Proc. Natl. Acad. Sci. U S A 99, 14512–14517. 10.1073/pnas.222546399 12391303 PMC137914

[B23] Davies C. H. Davies S. N. Collingridge G. L. (1990). Paired-pulse depression of monosynaptic GABA-mediated inhibitory postsynaptic responses in rat hippocampus. J. Physiol. 424, 513–531. 10.1113/jphysiol.1990.sp018080 2167975 PMC1189826

[B24] Davies C. H. Starkey S. J. Pozza M. F. Collingridge G. L. (1991). GABA autoreceptors regulate the induction of LTP. Nature 349, 609–611. 10.1038/349609a0 1847993

[B25] Debanne D. Guérineau N. C. Gähwiler B. H. (1996). Paired-pulse facilitation and depression at unitary synapses in rat hippocampus: quantal fluctuation affects subsequent release. J. Physiol. 491, 163–176. 10.1113/jphysiol.1996.sp021204 9011608 PMC1158767

[B26] Edelmann E. Cepeda-Prado E. Franck M. Lichtenecker P. Brigadski T. Leßmann V. (2015). Theta burst firing recruits BDNF release and signaling in postsynaptic CA1 neurons in spike-timing-dependent LTP. Neuron 86, 1041–1054. 10.1016/j.neuron.2015.04.007 25959732

[B27] Engel J. Jr. da Silva F. L. (2012). High-frequency oscillations–where we are and where we need to go. Prog. Neurobiol. 98, 316–318. 10.1016/j.pneurobio.2012.02.001 22342736 PMC3374035

[B28] Fiumelli H. Woodin M. A. (2007). Role of activity-dependent regulation of neuronal chloride homeostasis in development. Curr. Opin. Neurobiol. 17, 81–86. 10.1016/j.conb.2007.01.002 17234400

[B29] Freund T. F. Katona I. (2007). Perisomatic inhibition. Neuron 56, 33–42. 10.1016/j.neuron.2007.09.012 17920013

[B30] Fries P. Nikolić D. Singer W. (2007). The gamma cycle. Trends Neurosci. 30, 309–316. 10.1016/j.tins.2007.05.005 17555828

[B31] Hebb D. O. (1949). The Organization of Behavior: A Neurophysiological Theory. New York: John Wiley and Sons.

[B32] Hess G. Gustafsson B. (1990). Changes in field excitatory postsynaptic potential shape induced by tetanization in the CA1 region of the guinea-pig hippocampal slice. Neuroscience 37, 61–69. 10.1016/0306-4522(90)90192-7 1978743

[B33] Huerta P. T. Lisman J. E. (1995). Bidirectional synaptic plasticity induced by a single burst during cholinergic theta oscillation in CA1 *in vitro*. Neuron 15, 1053–1063. 10.1016/0896-6273(95)90094-2 7576649

[B34] Jester J. M. Campbell L. W. Sejnowski T. J. (1995). Associative EPSP–spike potentiation induced by pairing orthodromic and antidromic stimulation in rat hippocampal slices. J. Physiol. 484, 689–705. 10.1113/jphysiol.1995.sp020696 7623285 PMC1157953

[B35] Kaila K. Price T. J. Payne J. A. Puskarjov M. Voipio J. (2014a). Cation-chloride cotransporters in neuronal development, plasticity and disease. Nat. Rev. Neurosci. 15, 637–654. 10.1038/nrn3819 25234263 PMC4294553

[B36] Kaila K. Ruusuvuori E. Seja P. Voipio J. Puskarjov M. (2014b). GABA actions and ionic plasticity in epilepsy. Curr. Opin. Neurobiol. 26, 34–41. 10.1016/j.conb.2013.11.004 24650502

[B37] Kang H. Welcher A. A. Shelton D. Schuman E. M. (1997). Neurotrophins and time: different roles for TrkB signaling in hippocampal long-term potentiation. Neuron 19, 653–664. 10.1016/s0896-6273(00)80378-5 9331355

[B38] Korte M. Carroll P. Wolf E. Brem G. Thoenen H. Bonhoeffer T. (1995). Hippocampal long-term potentiation is impaired in mice lacking brain-derived neurotrophic factor. Proc. Natl. Acad. Sci. U S A 92, 8856–8860. 10.1073/pnas.92.19.8856 7568031 PMC41066

[B39] Korte M. Staiger V. Griesbeck O. Thoenen H. Bonhoeffer T. (1996). The involvement of brain-derived neurotrophic factor in hippocampal long-term potentiation revealed by gene targeting experiments. J. Physiol. Paris 90, 157–164. 10.1016/s0928-4257(97)81415-5 9116659

[B40] Larson J. Lynch G. (1986). Induction of synaptic potentiation in hippocampus by patterned stimulation involves two events. Science 232, 985–988. 10.1126/science.3704635 3704635

[B41] Larson J. Munkácsy E. (2015). Theta-burst LTP. Brain Res. 1621, 38–50. 10.1016/j.brainres.2014.10.034 25452022 PMC4411212

[B42] Larson J. Wong D. Lynch G. (1986). Patterned stimulation at the theta frequency is optimal for the induction of hippocampal long-term potentiation. Brain Res. 368, 347–350. 10.1016/0006-8993(86)90579-2 3697730

[B43] Lisman J. E. Jensen O. (2013). The θ-γ neural code. Neuron 77, 1002–1016. 10.1016/j.neuron.2013.03.007 23522038 PMC3648857

[B44] Lu Y. M. Mansuy I. M. Kandel E. R. Roder J. (2000). Calcineurin-mediated LTD of GABAergic inhibition underlies the increased excitability of CA1 neurons associated with LTP. Neuron 26, 197–205. 10.1016/s0896-6273(00)81150-2 10798404

[B45] Maccaferri G. Lacaille J. C. (2003). Interneuron diversity series: hippocampal interneuron classifications-making things as simple as possible, not simpler. Trends Neurosci. 26, 564–571. 10.1016/j.tins.2003.08.002 14522150

[B46] Manabe T. Nicoll R. A. (1994). Long-term potentiation: evidence against an increase in transmitter release probability in the CA1 region of the hippocampus. Science 265, 1888–1892. 10.1126/science.7916483 7916483

[B47] Manabe T. Wyllie D. J. Perkel D. J. Nicoll R. A. (1993). Modulation of synaptic transmission and long-term potentiation: effects on paired pulse facilitation and EPSC variance in the CA1 region of the hippocampus. J. Neurophysiol. 70, 1451–1459. 7904300 10.1152/jn.1993.70.4.1451

[B48] Mann E. O. Paulsen O. (2007). Role of GABAergic inhibition in hippocampal network oscillations. Trends Neurosci. 30, 343–349. 10.1016/j.tins.2007.05.003 17532059

[B49] Marder C. P. Buonomano D. V. (2003). Differential effects of short- and long-term potentiation on cell firing in the CA1 region of the hippocampus. J. Neurosci. 23, 112–121. 12514207 10.1523/JNEUROSCI.23-01-00112.2003 PMC6742129

[B50] Markram H. Lübke J. Frotscher M. Roth A. Sakmann B. (1997). Physiology and anatomy of synaptic connections between thick tufted pyramidal neurones in the developing rat neocortex. J. Physiol. 500, 409–440. 10.1113/jphysiol.1997.sp022031 9147328 PMC1159394

[B51] McNaughton B. L. (1980). Evidence for two physiologically distinct perforant pathways to the fascia dentata. Brain Res. 199, 1–19. 10.1016/0006-8993(80)90226-7 7407615

[B52] McNaughton B. L. (1982). Long-term synaptic enhancement and short-term potentiation in rat fascia dentata act through different mechanisms. J. Physiol. 324, 249–262. 10.1113/jphysiol.1982.sp014110 7097600 PMC1250703

[B53] Mehta M. R. Lee A. K. Wilson M. A. (2002). Role of experience and oscillations in transforming a rate code into a temporal code. Nature 417, 741–746. 10.1038/nature00807 12066185

[B54] Nicoll R. A. Alger B. E. (1979). Presynaptic inhibition: transmitter and ionic mechanisms. Int. Rev. Neurobiol. 21, 217–258. 10.1016/s0074-7742(08)60639-x 43844

[B55] Nishida H. Takahashi M. Lauwereyns J. (2014). Within-session dynamics of theta-gamma coupling and high-frequency oscillations during spatial alternation in rat hippocampal area CA1. Cogn. Neurodyn. 8, 363–372. 10.1007/s11571-014-9289-x 25206930 PMC4155064

[B56] O’Keefe J. Dostrovsky J. (1971). The hippocampus as a spatial map. Preliminary evidence from unit activity in the freely-moving rat. Brain Res. 34, 171–175. 10.1016/0006-8993(71)90358-1 5124915

[B57] O’Keefe J. Recce M. L. (1993). Phase relationship between hippocampal place units and the EEG theta rhythm. Hippocampus 3, 317–330. 10.1002/hipo.450030307 8353611

[B58] Paulsen O. Sejnowski T. J. (2006). From invertebrate olfaction to human cognition: emerging computational functions of synchronized oscillatory activity. J. Neurosci. 26, 1661–1662. 10.1523/jneurosci.3737-05a.2006 16467511 PMC2911952

[B59] Pearce R. A. (1993). Physiological evidence for two distinct GABA_A_ responses in rat hippocampus. Neuron 10, 189–200. 10.1016/0896-6273(93)90310-n 8382497

[B60] Pugliese A. M. Ballerini L. Passani M. B. Corradetti R. (1994). EPSP-spike potentiation during primed burst-induced long-term potentiation in the CA1 region of rat hippocampal slices. Neuroscience 62, 1021–1032. 10.1016/0306-4522(94)90340-9 7845583

[B61] Ranck J. B. (1973). Studies on single neurons in dorsal hippocampal formation and septum in unrestrained rats. I. Behavioral correlates and firing repertoires. Exp. Neurol. 41, 461–531. 10.1016/0014-4886(73)90290-2 4355646

[B62] Schulz P. E. Cook E. P. Johnston D. (1995). Using paired-pulse facilitation to probe the mechanisms for long-term potentiation (LTP). J. Physiol. Paris 89, 3–9. 10.1016/0928-4257(96)80546-8 7581296

[B63] Sejnowski T. J. Paulsen O. (2006). Network oscillations: emerging computational principles. J. Neurosci. 26, 1673–1676. 10.1523/jneurosci.3737-05d.2006 16467514 PMC2915831

[B64] Semyanov A. Walker M. C. Kullmann D. M. Silver R. A. (2004). Tonically active GABA_A_ receptors: modulating gain and maintaining the tone. Trends Neurosci. 27, 262–269. 10.1016/j.tins.2004.03.005 15111008

[B65] Singer W. Gray C. M. (1995). Visual feature integration and the temporal correlation hypothesis. Annu. Rev. Neurosci. 18, 555–586. 10.1146/annurev.neuro.18.1.555 7605074

[B66] Smith J. Lal V. Bowser D. Cappai R. Masters C. Ciccotosto G. (2009). Stimulus pattern dependence of the Alzheimer“s disease amyloid-beta 42 peptide’s inhibition of long term potentiation in mouse hippocampal slices. Brain Res. 1269, 176–184. 10.1016/j.brainres.2009.03.007 19302985

[B67] Song S. Miller K. D. Abbott L. F. (2000). Competitive Hebbian learning through spike-timing-dependent synaptic plasticity. Nat. Neurosci. 3, 919–926. 10.1038/78829 10966623

[B68] Staff N. P. Spruston N. (2003). Intracellular correlate of EPSP-spike potentiation in CA1 pyramidal neurons is controlled by GABAergic modulation. Hippocampus 13, 801–805. 10.1002/hipo.10129 14620875

[B69] Stent G. S. (1973). A physiological mechanism for Hebb’s postulate of learning. Proc. Natl. Acad. Sci. U S A 70, 997–1001. 10.1073/pnas.70.4.997 4352227 PMC433410

[B70] Suh J. Rivest A. J. Nakashiba T. Tominaga T. Tonegawa S. (2011). Entorhinal cortex layer III input to the hippocampus is crucial for temporal association memory. Science 334, 1415–1420. 10.1126/science.1210125 22052975

[B71] Thompson L. T. Best P. J. (1989). Place cells and silent cells in the hippocampus of freely-behaving rats. J. Neurosci. 9, 2382–2390. 2746333 10.1523/JNEUROSCI.09-07-02382.1989 PMC6569761

[B72] Tiesinga P. Fellous J. M. Sejnowski T. J. (2008). Regulation of spike timing in visual cortical circuits. Nat. Rev. Neurosci. 9, 97–107. 10.1038/nrn2315 18200026 PMC2868969

[B76] Tominaga Y. Ichikawa M. Tominaga T. (2009). Membrane potential response profiles of CA1 pyramidal cells probed with voltage-sensitive dye optical imaging in rat hippocampal slices reveal the impact of GABA(A)-mediated feed-forward inhibition in signal propagation. Neurosci. Res. 64, 152–161. 10.1016/j.neures.2009.02.007 19428695

[B73] Tominaga T. Tominaga Y. (2010). GABA_A_ receptor-mediated modulation of neuronal activity propagation upon tetanic stimulation in rat hippocampal slices. Pflugers Arch. 460, 875–889. 10.1007/s00424-010-0870-9 20734201

[B74] Tominaga T. Tominaga Y. Ichikawa M. (2002). Optical imaging of long-lasting depolarization on burst stimulation in area CA1 of rat hippocampal slices. J. Neurophysiol. 88, 1523–1532. 10.1152/jn.00554.2001 12205172

[B75] Tominaga T. Tominaga Y. Yamada H. (2000). Quantification of optical signals with electrophysiological signals in neural activities of Di-4-ANEPPS stained rat hippocampal slices. J. Neurosci. Methods 102, 11–23. 10.1016/s0165-0270(00)00270-3 11000407

[B77] Wakita M. Kotani N. Kogure K. Akaike N. (2014). Inhibition of excitatory synaptic transmission in hippocampal neurons by levetiracetam involves Zn^2+^-dependent GABA type A receptor-mediated presynaptic modulation. J. Pharmacol. Exp. Ther. 348, 246–259. 10.1124/jpet.113.208751 24259680

[B78] Watanabe S. Hoffman D. A. Migliore M. Johnston D. (2002). Dendritic K^+^ channels contribute to spike-timing dependent long-term potentiation in hippocampal pyramidal neurons. Proc. Natl. Acad. Sci. U S A 99, 8366–8371. 10.1073/pnas.122210599 12048251 PMC123073

[B79] White J. A. Banks M. I. Pearce R. A. Kopell N. J. (2000). Networks of interneurons with fast and slow gamma-aminobutyric acid type A (GABA_A_) kinetics provide substrate for mixed gamma-theta rhythm. Proc. Natl. Acad. Sci. U S A 97, 8128–8133. 10.1073/pnas.100124097 10869419 PMC16681

[B80] Yamamoto J. Suh J. Takeuchi D. Tonegawa S. (2014). Successful execution of working memory linked to synchronized high-frequency gamma oscillations. Cell 157, 845–857. 10.1016/j.cell.2014.04.009 24768692

[B81] Zhu G. Liu Y. Wang Y. Bi X. Baudry M. (2015). Different patterns of electrical activity lead to long-term potentiation by activating different intracellular pathways. J. Neurosci. 35, 621–633. 10.1523/JNEUROSCI.2193-14.2015 25589756 PMC4293414

